# Transcriptome Responses to Dexamethasone Depending on Dose and Glucocorticoid Receptor Sensitivity in the Liver

**DOI:** 10.3389/fgene.2019.00559

**Published:** 2019-06-12

**Authors:** Eduard Murani, Nares Trakooljul, Frieder Hadlich, Siriluck Ponsuksili, Klaus Wimmers

**Affiliations:** Institute for Genome Biology – Leibniz Institute for Farm Animal Biology, Dummerstorf, Germany

**Keywords:** dexamethasone, glucocorticoid receptor, glucocorticoid sensitivity, liver, protocadherin, transcriptome

## Abstract

Tissue sensitivity to glucocorticoids is a key factor dictating outcome of their homeostatic and therapeutic action, whereby liver represents one of the major peripheral targets. Here, we used pigs carrying a natural gain-of-function glucocorticoid receptor (GR) variant Ala610Val (GR_Ala610Val_) as a model to identify genes and pathways related to differential glucocorticoid sensitivity. Animals with different GR_Ala610Val_ genotypes were treated either with saline or two different doses of dexamethasone. Genome-wide transcriptional responses depending on treatment, genotype, and their interaction in the liver were investigated using mRNA sequencing. Dexamethasone induced vast transcriptional responses, with more than 30% of present genes being affected. Functional annotation of genes differentially expressed due to dexamethasone treatment suggested that genes related to inflammation respond more sensitively, despite absence of an immune stimulus. In contrast, genes involved in glucose metabolism and cancer appeared to be less sensitive. Analysis of genotype and genotype × treatment interaction revealed that clustered protocadherins, particularly *PCDHB7*, are most prominently affected by GR_Ala610Val_, mainly depending on dose. GR_Ala610Val_ influenced also expression of a set of glucose metabolism related genes, including *PPARGC1A* and *CEBPB*, in the absence of dexamethasone though no differences in basal plasma glucose level were observed. This might represent an adaptive response, keeping balance between receptor sensitivity, and level of circulating endogenous glucocorticoids. Administration of low dexamethasone dose changed their expression pattern and induced higher glucose response in carriers of the hypersensitive Val receptor. Our findings suggest that GR_Ala610Val_ modulates tissue responses to glucocorticoids dynamically, depending on their circulating level.

## Introduction

Glucocorticoid receptor (GR) signaling is a subject of intense research because of its vital role in prenatal development, stress response, and as an important drug target in human and veterinary medicine, primarily due to its immune-modulatory actions (Kadmiel and Cidlowski, 2013; Wyns et al., 2013). GR is a ligand activated transcription factor that controls gene expression in a mechanistically complex, and context dependent manner (Weikum et al., 2017). Genomic responses triggered by GR activation are fine-tuned at multiple, interconnected levels. These include ligand (i.e., glucocorticoid) production and bioavailabiliy, alternative processing of GR gene (*NR3C1*) and protein, and its interactions with coregulators and with the chromatin landscape (Sacta et al., 2016; Weikum et al., 2017). Genetic and epigenetic variation, including GR mutations, may affect each of these regulatory mechanisms and cause inter-individual differences in responses to natural glucocorticoids (GC) as well as GC-based pharmacotherapy (Quax et al., 2013). Knowledge of GR target genes and networks and better understanding of molecular mechanisms controlling its genomic actions may thus on one hand help to develop more targeted GC-therapies (Phuc Le et al., 2005), but also provide insight into pathobiology of disorders resulting from dysregulated natural GC signaling (Kadmiel and Cidlowski, 2013).

Because gene networks governed by GR are largely unknown in farm animals, the aim of the present study was to explore genome-wide transcriptome responses to GR activation in the pig liver using mRNA-seq. Liver is a major target for homeostatic actions of GR. Regulation of glucose homeostasis is a well-recognized function of GR in the liver (Patel et al., 2014), but experiments in model organisms indicate that hepatic GR is also involved in local and systemic control of inflammatory response, and feedback control of GC bioavailability (Quinn and Cidlowski, 2016; Jenniskens et al., 2018). So far GR-regulated trancriptome (Phuc Le et al., 2005; Reddy et al., 2007; Frijters et al., 2010) and regulatory mechanisms (Grøntved et al., 2013; Lim et al., 2015) in the liver were studied on a genome-wide scale essentially only in rodents. We explored the transcriptome GC responses in the context of a unique natural gain-of-function substitution Ala610Val in the porcine GR (GR_Ala610Val_) (Murani et al., 2012; Reyer et al., 2016). Different from GR deficiency or resistance, GR hypersensitivity has not been well researched yet (Nicolaides and Charmandari, 2017). We showed previously that animals carrying the GR_Ala610Val_ substitution feature profound reduction in the activity of the hypothalamus-pituitary-adrenocortical (HPA) axis and ultimately GC production since early ontogeny yet are otherwise phenotypically normal under resting conditions (Muráni et al., 2016). While this observation evidences compensatory changes at the level of ligand production, it is not known if, and how, other levels of GR signaling are influenced by GC hypersensitivity of the mutated receptor. Thus, the second aim of this study was to obtain better understanding of the molecular and phenotypic consequences of GR_Ala610Val_, including its effect on sensitivity to application of exogenous GC, by examining its transcriptome signature in untreated and GC-treated animals. To activate GR we treated the animals with dexamethasone, a synthetic GC widely used as an anti-inflammatory drug. Dexamethasone is a selective GR agonist, and is not bound by corticosteroid binding globulin (CBG), thus triggers specific responses. We administered dexamethasone at two doses (60 μg/kg as prescribed, and a lower dose of 10 μg/kg live weight), because with increasing doses inter-individual differences in GC sensitivity and responses diminish (Chriguer et al., 2005; Muráni et al., 2016). This allowed us to investigate also dose-sensitivity in the transcriptional responses to GR activation, which was to our knowledge not examined previously, at least not in the liver context.

## Results

### Metabolic Responses to Dexamethasone Treatment

**FIGURE 1:**
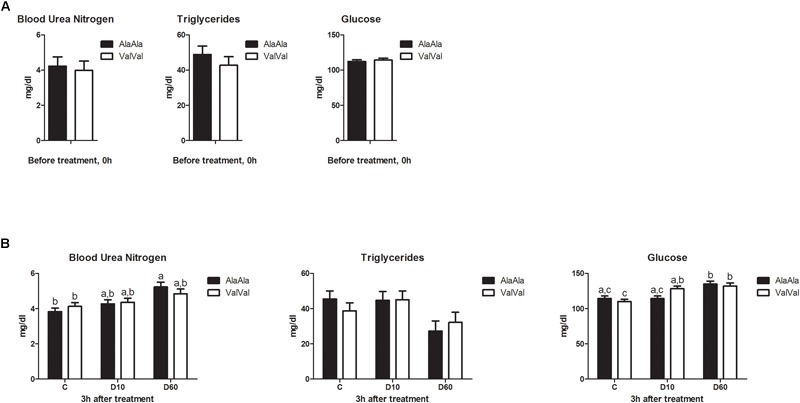
Effect of the GR_Ala610Val_ substitution on baseline metabolite levels **(A)** and on metabolic responses to dexamethasone treatment **(B)**. **(A)** Baseline metabolite levels in plasma before treatment (0 h). Each bar represents *n* = 24 per genotype group. **(B)** Metabolite levels in plasma 3 h after treatment. The different groups were treated with saline (C; each bar represents *n* = 10 per genotype group), 10 μg/kg dexamethasone (D10; each bar represents *n* = 8 per genotype group), or 60 μg/kg dexamethasone (D60; each bar represents *n* = 6 per genotype group), respectively. Results are presented as least-squares means + SEM. Bars sharing the same superscript are not significantly different at *p* < 0.05. For Glucose main effect of genotype × treatment interaction was significant at *p* < 0.05.

GR_Ala610Val_ genotype showed no significant effect on baseline levels of any of the metabolic parameters at 0h (Figure 1A). Treatment had significant effect on BUN (*p* < 0.001), TG (*p* = 0.016), and Glu (*p* < 0.001) levels at 3 h (Figure 1B). Genotype showed no significant effect on any of the metabolic parameters across treatments at 3 h, but we found significant interaction between genotype and treatment for Glu (*p* = 0.026). D10 significantly increased Glu levels in ValVal but not in AlaAla animals (Figure 1B), which is in line with the expected higher glucocorticoid sensitivity of the Val variant.

### Genome-Wide Transcriptional Responses to Dexamethasone Treatment

**FIGURE 2:**
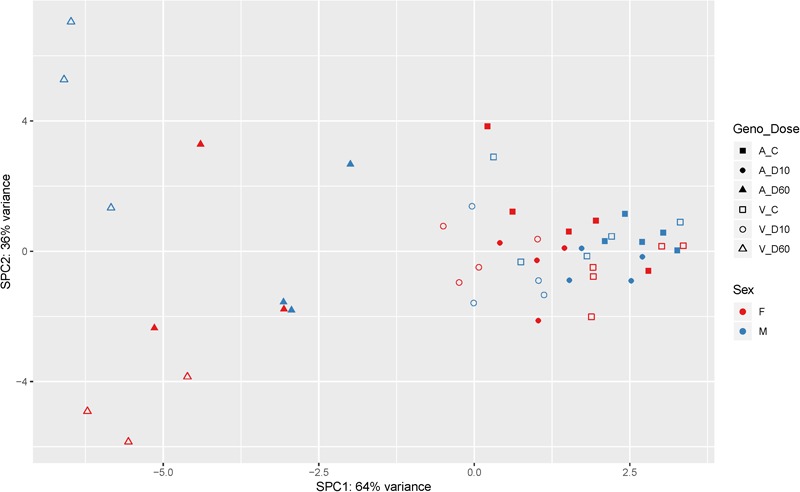
Supervised principal component analysis of the mRNA-Seq data (cpm – counts per million; *n* = 48). Filled symbols indicate AlaAla genotype. Open symbols indicate ValVal genotype. Red coloring indicates females. Blue males, respectively. Squares indicate control saline treated group (C). Circles indicate 10 μg/kg dexamethasone treated group (D10), and triangles indicate 60 μg/kg dexamethasone treated group (D60).

**FIGURE 3:**
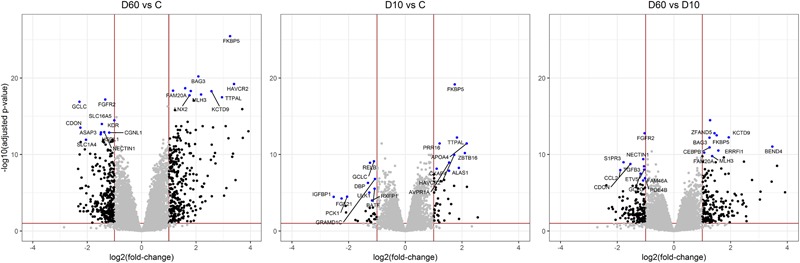
Volcano plots illustrating results of pairwise comparisons of gene expression between treatments (C, saline; D10, 10 μg/kg dexamethasone; D60, 60 μg/kg dexamethasone). To visualize the extent of the overall transcriptome changes due to treatment differentially expressed genes significant at *q* > 0.1 showing a fold change >2 are highlighted by black dots. The red lines display the respective thresholds on corresponding log scale. Ten most significantly up- or downregulated genes, respectively, are highlighted by blue dots and annotated with corresponding gene names.

After the filtering step (as outlined in the section “Materials and Methods”) data on the expression of 14179 genes were retained for the analysis of differentially expressed (DE) genes. Figure 2 shows the results of supervised principal component analysis (SPCA) of the data. This shows that the largest separation of the individual data was due to D60 treatment. The effect of dexamethasone treatment on liver transcriptome was analyzed in three pairwise comparisons: between the D60 and C groups (D60vsC), between the D10 and C groups (D10vsC), and between the D60 and D10 treatment groups (D60vsD10), respectively. At the FDR level of *q* ≤ 0.10 (*p* ≤ 0.039, 0.027, and 0.032, respectively) the analysis yielded 5558 (2840 up- and 2718 downregulated), 3874 (1988 up- and 1886 downregulated), and 4540 (2364 up- and 2176 downregulated) DE genes, respectively (results are summarized in Figure 3 and Supplementary Table S1. Overlap between the gene lists is summarized in Supplementary Figure S1). To obtain a comprehensive insight into biological functions and pathways influenced by dexamethasone treatment in the liver we first performed functional annotation of DE genes identified by the D60vsC comparison. The results are summarized in Supplementary Table S2. At the molecular and cellular level D60 treatment affected genes involved mainly in the regulation of cell growth, death, and maintenance, RNA expression, and macronutrient metabolism. Among these ingenuity pathway analysis (IPA) predicted negative regulation of various cellular functions related to homeostasis of blood cells. In line with the positive effect of GC on hepatic gluconeogenesis, IPA evidenced increased carbohydrate quantity [*z*-score = 2.56, -log(B-H *p*-value) = 7.43]. Other predicted metabolic consequences included decreased synthesis of fatty acids [*z*-score = -2.09, -log(B-H *p*-value) = 5.28] and protein breakdown [*z*-score = -2.54, -log(B-H *p*-value) = 8.92]. The transcriptional response to dexamethasone showed highly significant association with liver steatosis [-log(B-H *p*-value) = 10.1], which is well-established side effect of GC therapy (Nader et al., 2010; Kadmiel and Cidlowski, 2013), however, no specific direction could be deduced (z-score = 0.19). As expected, one of the top enriched canonical pathways revealed by IPA was the glucocorticoid signaling pathway [-log(B-H *p*-value) = 6.26]. In fact, *FKBP5*, a key member of the pathway participating in the regulation of GR sensitivity (Kästle et al., 2018), was the most significant DE gene (*q* = 3.4E–26E; Figure 3), emphasizing the importance of tight regulation of glucocorticoid signaling. Consistent with the deduced molecular and physiological actions of dexamethasone treatment, IPA predicted i.a. activation of PPAR [*z*-score = 2.71, -log(B-H *p*-value) = 4.9], and inhibition of AMPK [*z*-score = -2.29, -log(B-H *p*-value) = 6.07], NFKB [*z*-score = -3.73, -log(B-H *p*-value) = 4.90], and ERK/MAPK [*z*-score = -3.83, -log(B-H *p*-value) = 4.42] signaling pathways. Furthermore, IPA correctly identified dexamethasone (and other GC such as methylprednisolone) as activated upstream regulator [*z*-score = 3.02, -log(*p*-value) = 2.68]. The most significant transcriptional regulator was the transcription factor HNF4A [-log(*p*-value) = 32], which is a pivotal mediator of glucocorticoid signaling in the liver (Reddy et al., 2007). Other suggested prominent upstream regulatory events were, for example, activation of MYC [*z*-score = 4.68, -log(*p*-value) = 3.82] and KRAS [*z*-score = 2.01, -log(*p*-value) = 7.95], and inhibition of TNF [*z*-score = -3.43, -log(*p*-value) = 9.18] or FOXM1 [*z*-score = -3.30, -log(*p*-value) = 3.50].

**FIGURE 4:**
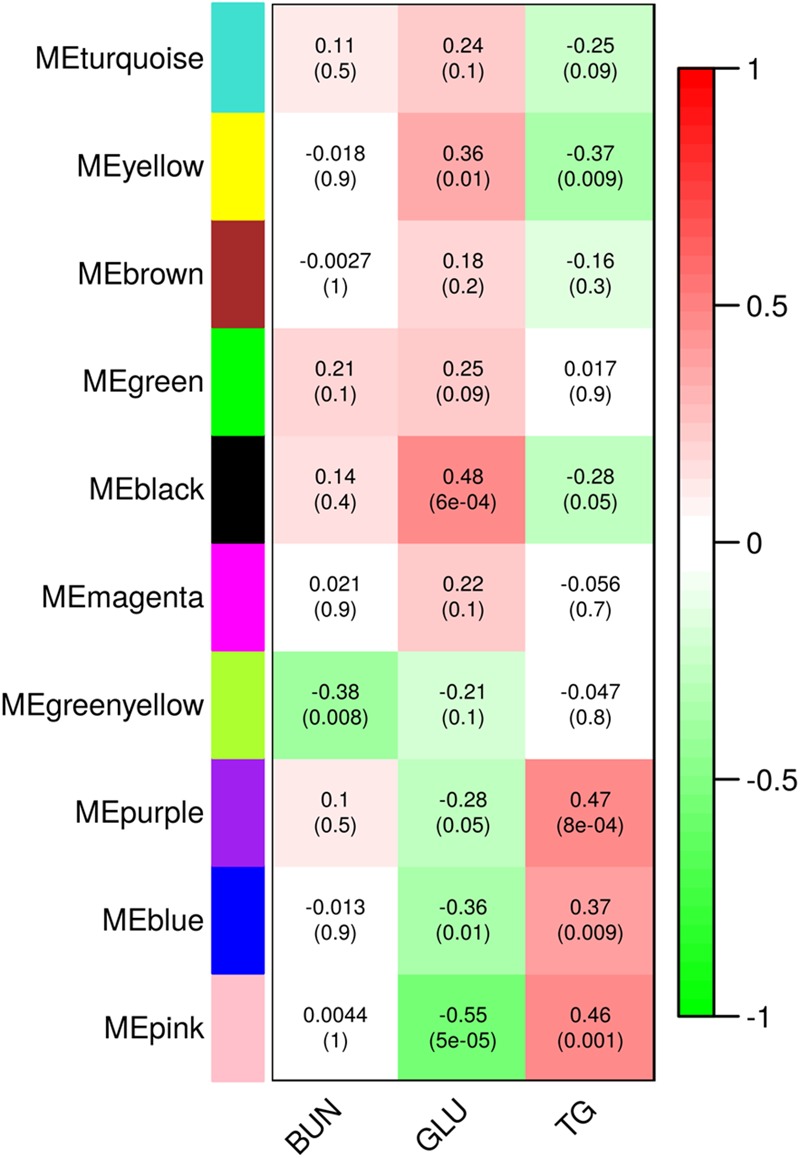
Modules of co-regulated genes identified using weighted correlation network analysis (WGCNA). Values in the heatmap show correlation of module eigenvalues with metabolite levels in plasma 3 h following dexamethasone treatment. Upper values show Pearson correlation coefficients and lower values in parenthesis show the corresponding *p*-values. BUN, blood urea nitrogen; GLU, glucose; TG, triglycerides.

In order to relate the metabolic and transcriptional responses to dexamethasone application we performed weighted correlation network analysis (WGCNA) for all DE genes compared to control (DE in D60vsC or D10vC, respectively; in total 7030 genes). WGCNA revealed ten modules of co-expressed genes (Figure 4 and Supplementary Table S1). The eigenvalues of three modules, designated pink, blue and purple, correlated negatively with Glu and positively with TG level. Modules black and yellow showed an opposite pattern. Only the module greenyellow showed significant correlation with BUN. For module black, displaying the strongest positive relationship with Glu, no significantly enriched canonical pathways could be found using IPA after *p*-value (BH) adjustment. Nevertheless, several key GR coregulators involved in gluconeogenesis belonged to module black, including *HNF4A, FOXO1, FOXO3, FOXA2, CEBPB*, and *PPARGC1A*. In the module pink, displaying the strongest negative relationship with Glu, the top enriched canonical pathway was related to Wnt/β-catenin signaling. Though typically associated with embryogenesis and development, this pathway is also implicated in the modulation of hepatic glucose metabolism (Liu et al., 2011). Pathway annotation of the six modules of co-expressed gene related to metabolic responses, reaching at least nominal significance, is summarized in Supplementary Table S3.

### Biological Context of Dose-Dependent Transcriptional Regulation by Dexamethasone

In the next step we sought to explore which biological functions and regulatory circuits are associated with differential glucocorticoid sensitivity at the transcriptional level. Therefore we categorized genes according to their sensitivity to dexamethasone treatment indicated by the pattern of differential expression between the three treatment groups, and compared functional gene enrichment using IPA. We focused on two sets of genes; those showing consistent dose-dependent (DD) transcriptional response to dexamethasone (significantly differentially expressed in all three pairwise comparisons, showing consistent direction of expression changes; in total 867 genes; Supplementary Table S1), and those with lower sensitivity (LS) responding only to D60 treatment (consistently differentially expressed in D60vsC and D60vsD10 comparisons, but not in D10vsC, with fold change < 1.1 in the latter; in total 1580 genes; Supplementary Table S1). In Supplementary Figure S2, clustering of samples based on expression profiles of top 100 DD genes is presented. The samples cluster according to treatment demonstrating suitability of DD genes as biomarkers of GC action.

With regards to molecular and cellular functions the most obvious difference showed genes associated with increased carbohydrate quantity, which were significantly enriched only in the LS set [*z*-score = 3.81, -log(*p*-value) = 2.56], and with protein ubiquitination which were significantly enriched solely in the DD set [*z*-score = 2.52, -log(*p*-value) = 2.84] (Supplementary Table S4). Both sets were enriched for genes involved in homeostasis of blood cells (leukocytes), but the DD set alone showed significant evidence for a negative effect [*z*-score = -2.24, -log(*p*-value) = 2.63]. Furthermore, the LS gene set is associated with inhibition of liver cancer [*z*-score = -2.8, -log(*p*-value) = 2.59]. Only genes in the DD set were significantly enriched for those belonging to canonical GR signaling pathway [-log(B-H *p*-value) = 1.3] and were predicted by IPA to be regulated by dexamethasone [*z*-score = 2.12, -log(*p*-value) = 1.79]. Other canonical pathways significantly enriched exclusively in the DD set included for example PPAR signaling [*z*-score = 2.11, -log(B-H *p*-value) = 1.3]. Signaling pathways significantly enriched in the LS set included several growth factors [e.g., IGF1; *z*-score = -2.31, -log(*p*-value) = 1.53], and immune response-related pathways [e.g., NFKB; *z*-score = -1.96, -log(*p*-value) = 1.539]. In contrast, IPA predicted inhibition of several immune response regulators, in particular TNF [*z*-score = -3.78, -log(*p*-value) = 1.49] in the DD set, but not in the LS set. Several other regulators showing differential activation pattern among DD and LS genes are involved in cell proliferation and cancer, e.g., KRAS [*z*-score = 2.14, -log(*p*-value) = 3.15] predicted to be activated in DD, or FOXM1 inhibited in LS [*z*-score = -3.24, -log(*p*-value) = 2.98].

We complemented the IPA analysis of upstream regulators by analysis of over-represented binding motifs in upstream 2 kb regions using the oPOSSUM tool. The results are summarized in Supplementary Table S5. Among motifs emerging only in one of the gene sets was NFKB1, significantly over-represented in DD, and HNF1A, significantly over-represented in LS. This provides additional evidence that DD genes tend to be enriched for immune-related genes, while the LS set contains genes related to carbohydrate metabolism. Interestingly, GR (*NR3C1*) binding motif had a high (7.76), yet not significant, *z*-score in LS, but a very low *z*-score (0.328) in the DD set. This finding indicates distinct mode of regulation of genes in the two sets by GR.

### Differential Transcriptome Profiles Associated With GRAla610Val Depending on Treatment

To explore the impact of GR_Ala610Val_ on baseline liver transcriptome we analyzed DE genes due to genotype in the C group. A total of 251 genes (GinC set) was differentially expressed between AlaAla and ValVal at *p* < 0.05 (111 up- and 140 downregulated; Supplementary Table S6). Only one gene, *PCDHB7*, showed significant genotype effect after *p*-value adjustment [*q* = 0.004, -log(*p*-value) = 6.58]. To identify genes affected by genotype × treatment interaction we first compared expression changes induced by dexamethasone treatment between genotypes [difference D60 (or D10, respectively) vs. C in ValVal compared to D60 (or D10, respectively) vs. C in AlaAla]. Among genes showing differential response to treatment based on genotype (at *p* < 0.05) we further selected those differentially expressed between AlaAla and ValVal at *p* < 0.05 in either D10 or D60 and C group, respectively (Supplementary Table S6; GinD10 and GinD60, respectively). Using these criteria we found a total of 299 genes showing evidence (at *p* < 0.05) for genotype × treatment interaction for D10 (GxD10 set), and 326 for D60 (GxD60 set), respectively (Supplementary Table S6). About 50% of the genotype associated DE genes displayed also dexamethasone responsiveness (Supplementary Figure S3). Taken together, these data suggest, that GR_Ala610Val_ influences a subset of GR target genes, rather than inducing a general shift in GC responsiveness. Functional gene annotation using IPA (Supplementary Table S7) did not reveal significant enrichment after BH adjustment because of a relatively small number of DE genes. At nominal significance level top molecular and cellular functions enriched in the G set were primarily related to metabolism, including carbohydrate metabolism (23 genes). In the GxD60 and GxD10 sets in turn, cellular function and maintenance were the top functional themes (Supplementary Table S7). Remarkably, besides *PCDHB7* additional four protocadherins (*PCDHB5, PCDHGA7, PCDHGB4*, and *PCDHGB7*) were affected by genotype or genotype × treatment interaction.

### qPCR Validation of mRNA-Seq Results

To validate mRNA-Seq results we analyzed expression of three key GC response genes (*FKBP5, TSC22D3*, and *DUSP1*) and nine genes influenced by GR_Ala610Val_ genotype (*CEBPB, FOSL2, PCDHB7, PFKFB3, PPARGC1A, SCARA5, FAXDC2, GCK*, and *SLC17A3*) using qPCR. The qPCR expression patterns are presented in Supplementary Figure S4. For all genes the two data sets showed significant [-log(*p*-value) > 3) correlation with correlation coefficients ranging from *r* = 0.474 (*FAXDC2*) to 0.946 (*FKBP5*) (*r* = 0.850, 0.782, 0.503, 0.684, 0.668, 0.928, 0.705, 0.728, 0.679, and 0.485 for *TSC22D3, DUSP1, CEBPB, FOSL2, PCDHB7, PFKFB3, PPARGC1A, SCARA5, GCK*, and *SLC17A3*, respectively). A comparison of results for the effects of genotype and genotype × treatment interaction is presented in Table 1. For *PCDHB7, SCARA5*, and *GCK* significant genotype or genotype × treatment interaction were confirmed while for *PFKFB, PPARGC1A*, and *FAXDC2* similar tendency was observed. Overall these results demonstrate reliability of the mRNA-Seq analysis. The magnitude of the expression changes (on average ∼1.5 fold) corresponds with the changes in HPA axis activity, including glucocorticoid production, triggered by GR_Ala610Val_ (Muráni et al., 2016).

**Table 1:** Comparison of estimated genotype and genotype × treatment interaction effects of the GR_Ala610Val_ substitution on the expression of selected genes.

			Genotype (GinC)^1^		Genotype × D10 (G × D10)^2^		Genotype × D60 (G × D60)^3^	
Gene	Ensembl ID	Method	log2 FC^4^	*p*-value	log2 FC^4^	*p*-value	log2 FC^4^	*p*-value
*CEBPB*	ENSSSCG00000034207	RNA-seq	0.298	**0.027**	0.084	0.645	-0.024	0.921
		qPCR	0.117	0.760	-0.207	0.718	0.073	0.907
*FOSL2*	ENSSSCG00000032527	RNA-seq	0.438	**0.003**	-0.532	**0.010**	-0.296	0.226
		qPCR	0.181	0.340	0.039	0.890	-0.106	0.731
*PCDHB7*	ENSSSCG00000036961	RNA-seq	2.777	**<0.001**	-0.019	0.976	-2.526	**0.006**
		qPCR	0.934	**0.011**	-0.089	0.866	-1.091	0.063
*PFKFB3*	ENSSSCG00000011133	RNA-seq	-1.028	**0.006**	1.298	**0.011**	0.368	0.600
		qPCR	-0.844	0.065	1.177	0.085	0.411	0.575
*PPARGC1A*	ENSSSCG00000029275	RNA-seq	0.351	**0.013**	-0.344	0.075	-0.302	0.206
		qPCR	0.278	0.300	-0.159	0.691	-0.278	0.524
*SCARA5*	ENSSSCG00000009672	RNA-seq	0.492	**0.038**	-0.900	**0.008**	-0.757	0.059
		qPCR	0.742	**0.006**	-0.993	**0.013**	-1.134	**0.010**
*FAXDC2*	ENSSSCG00000017068	RNA-seq	-0.599	**0.016**	0.948	**0.006**	1.149	**0.010**
		qPCR	-0.595	0.151	0.941	0.091	0.893	0.139
*GCK*	ENSSSCG00000016751	RNA-seq	-1.102	**0.012**	1.225	**0.041**	-0.030	0.968
		qPCR	-1.233	**0.043**	1.414	0.077	0.779	0.365
*SLC17A3*	ENSSSCG00000037547	RNA-seq	0.476	**0.046**	-1.142	**0.001**	-1.018	0.055
		qPCR	0.291	0.443	-0.576	0.231	-0.611	0.243

*^*1*^Genotype (ValVal vs. AlaAla) effect on baseline expression estimated in the saline control (C) group. ^*2*^Genotype × treatment interaction estimated by contrasting D10 treatment (10 μg/kg dexamethasone) effects between the genotype groups (expression change between D10 vs. C in ValVal compared to D10 vs. C in AlaAla). ^*3*^Genotype × treatment interaction estimated by contrasting D60 treatment (60 μg/kg dexamethasone) effects between the genotype groups (expression change between D60 vs. C in ValVal compared to D60 vs. C in AlaAla). ^*4*^Log2 fold change differences between ValVal vs. AlaAla. Bold values highlight *p* < 0.05.*

We further independently validated the genotype effect on baseline hepatic expression of four genes of interest in a set of 80 liver samples from our previous study (Muráni et al., 2016). Results are presented in Table 2. For *PCDHB7* and *PPARGC1A* significant upregulation due to GR_Ala610Val_ was confirmed. For *SCARA5* GR_Ala610Val_ genotype showed opposite tendency. This might be related to the observation that GR_Ala610Val_ effect on *SCARA5* is depending on GC level, which in this set was elevated due to the sampling procedure. Expression of *PFKFB3* tended to be reduced by GR_Ala610Val_, as found in the main experiment, but only numerically.

**Table 2:** Effect of the GR_Ala610Val_ substitution on baseline hepatic expression of selected genes in an independent cohort.

		LSM ± SE^1^			
		AlaAla	AlaVal	ValVal	
Gene	ENSEMBL_ID	*n* = 18	*n* = 41	*n* = 21	*p*-value^2^
*PCDHB7*	ENSSSCG00000036961	8.01^a^ ± 0.16	8.31^a,b^ ± 0.10	8.55^b^ ± 0.15	**0.042**
*PFKFB3*	ENSSSCG00000011133	11.81 ± 0.29	11.86 ± 0.19	11.69 ± 0.28	0.886
*PPARGC1A*	ENSSSCG00000029275	12.80^a^ ± 0.17	13.13^a,b^± 0.11	13.43^b^ ± 0.16	**0.031**
*SCARA5*	ENSSSCG00000009672	12.09 ± 0.19	11.63 ± 0.12	11.61 ± 0.18	0.109

*^*1*^Estimated least square means **±** standard error of estimate; log2 transformed expression values. ^*2*^*p*-value of the main effect of genotype. Significant differences between LSM are indicated by different superscript within a row (a,b,c and *p* ≤ 0.05). Bold values highlight *p* < 0.05.*

## Discussion

### Glucocorticoid Responsive Genes and Physiological Pathways in Porcine Liver

Biology of stress resilience is receiving increasing attention due to the serious consequences for patients and society resulting from stress-related disorders (Pfau and Russo, 2015). The interest in the concept of stress resilience is growing also in farm animals because it addresses preservation of health as well as performance (Colditz and Hine, 2016). In this regard hepatic GR, by the virtue of integrating metabolic, endocrine and immune stress responses, is ideally situated to promote stress resilience. To provide knowledge base about the function and signaling of hepatic GR in farm animals we analyzed genome-wide transcriptome responses to dexamethasone treatment in the porcine liver. The overall picture of biological functions influenced by dexamethasone (D60vsC) corresponds with previously reported GC responses in mice (Phuc Le et al., 2005; Frijters et al., 2010). However, there were some notable differences. For instance, as shown by SPCA in Figure 2, other than in mice (e.g., Frijters et al., 2010), the effect of sex was less pronounced compared to the effect of dexamethasone. One factor that might have contributed to this difference is that pigs used in the present study were not sexually mature. Another striking finding in our study was downregulation of *PCK1* expression in dexamethasone treated pigs (by both doses). *PCK1* encodes *PEPCK*, a rate-limiting enzyme in gluconeogenesis. Due to its key role in this process, regulation of *PCK1* is well studied, particularly in rodents. Expression of *PCK1* is reportedly activated by GR, both directly and by recruiting other coregulators involved in gluconeogenesis (Vegiopoulos and Herzig, 2007; Sacta et al., 2016). Species specific regulation of *PCK1* provides one likely explanation for the conflicting findings (Niu et al., 2018). Nevertheless we cannot exclude contribution of other factors like differences in the treatment (in the study of Frijters et al. (2010) mice were treated using a less potent GC methylprednisolone) and feeding state of the animals (see below). Different from *PCK1*, other genes encoding key enzymes involved in gluconeogenesis (*G6PC* and *PC*) were upregulated by dexamethasone (D60), evidencing a different mode of their regulation in this specific context. Several transcription factors and cofactors playing important role in gluconeogenesis (e.g., *HNF4A, KLF15, FOXO1, FOXO3, FOXA2, CEBPB*, and *PPARGC1A*) (Goldstein and Hager, 2015; Sacta et al., 2016) were upregulated by dexamethasone as well. However, similar to *G6PC* and *PC*, most of them (apart from *HNF4A* and *FOXO3*) were significantly induced only by D60. This, together with the functional annotation of DD and LS genes, points to lower dexamethasone sensitivity of genes related to glucose metabolism. One factor that might have contributed to this lower sensitivity is feeding state (all pigs were fed *ad libitum* during the experiment), and accordingly signaling of insulin, glucagon, and of glucose itself that modulate GR action on gluconeogenic genes (Meyer et al., 1991; Pierreux et al., 1999; Vegiopoulos and Herzig, 2007). In fact, a recent study by Kalvisa et al. (2018) demonstrated that the impact of feeding on hepatic gene expression is depending on cooperate action of GR and insulin signaling pathways. The authors showed that feeding significantly reduces occupancy of chromatin by GR. While this is partly due to reduced glucocorticoid concentration following feeding, only a subset of feeding repressed genes was activated by preprandial dexamethasone application (Kalvisa et al., 2018), evidencing that insulin indeed interferes with GR signaling to some extent.

Noticeably, in our study *CREB1*, a key transcription factor in gluconeogenesis mediating glucagon action, was downregulated by both dexamethasone doses. Goldstein et al. (2017) demonstrated that whereas GR and CREB1 synergize on *PCK1* promoter, CREB1 binding is not augmented by GR on *G6PC* promoter in mice, providing potential explanation for different responses of *PCK1*, and *G6PC* observed here. Unfortunately, previous expression studies focused on mice (Phuc Le et al., 2005; Frijters et al., 2010; Kalvisa et al., 2018) and thus information about the impact of dexamethasone on hepatic *CREB1* expression in other species is limited.

Another biological and therapeutic function of dexamethasone that seems to require higher doses is suppression of cell proliferation and cancer. Accordingly, IPA predicted inhibition of FOXM1 signaling in LS genes. Aberrant expression of FOXM1 plays an essential role in hepatocellular carcinoma (Yu et al., 2016), however, *FOXM1* itself was only numerically reduced by D60, implying either post-transcriptional regulation or indirect involvement of FOXM1.

In contrast to glucose metabolism or cancer-related pathways, our results suggested higher dexamethasone sensitivity of immune related pathways, and despite absence of an immune stimulus. Indeed, key anti-inflammatory mediators induced by GR, such as *DUSP1* and *TSC22D3* (GILZ) (Coutinho and Chapman, 2011), were among the top DD genes.

It is interesting to note that the analysis of transcription factor binding sites in 5′ flanking regions pointed to direct regulation of genes with lower dexamethasone sensitivity by GR, whereas genes responding dose-dependently appear to lack canonical GR binding sites (GRE). King et al. (2013) demonstrated that inflammatory genes regulated (repressed) by GR indirectly, were more potently repressed by dexamethasone compared to genes regulated by GR directly. High resolution mapping of GR binding sites using ChIP-exo in mice revealed that exogenous pharmacologic GC cause redistribution of GR in the liver from monomeric sites, occupied under physiological conditions, to dimeric binding sites. The gained dimeric sites are enriched for loci involved in glucose metabolism (Lim et al., 2015). Thus DD genes might be preferentially regulated by GR monomers in cooperation with other cofactors, while for LS genes binding of GR dimers to canonical GREs might be more common.

### Transcriptional Signature of GRAla610Val in the Liver

Our previous molecular and phenotypic characterization of GR_Ala610Val_ clearly demonstrated that the substitution causes hypersensitivity at the receptor level (Murani et al., 2012; Reyer et al., 2016). In addition, *ex-vivo* dexamethasone treatment of mitogen stimulated peripheral blood mononuclear cells (PBMC) indicated higher tissue GC sensitivity of GR_Ala610Val_ carriers (Muráni et al., 2016). Here we tested for the first time the hypothesis that GR_Ala610Val_ carriers are more sensitive to exogenous GC *in vivo*. The observation, that D10 significantly increased Glu levels only in ValVal animals, supports this notion. However, the fact, that at D60 no significant differences were observed illustrates difficulty to uncover and explore GC hypersensitivity *in vivo*. This implies that diagnosis of GC hypersensitivity requires testing of a wider range of different doses. A further limitation is that GR action is highly context specific, thus GC hypersensitivity may manifest only for a specific tissue or in a specific physiological state.

While GR_Ala610Val_ apparently influenced GC sensitivity of the liver, its transcriptome signature was rather modest, and involves only a subset of GR target genes. Strikingly, although basal Glu levels are not significantly affected by GR_Ala610Val_, we found several DE genes involved in carbohydrate metabolism in untreated animals. These include i.a. *PPARGC1A* and *CEBPB*. As mentioned above, both represent important coregulators of GR in the liver, and are themselves GC-responsive. In line with the evidence for higher GC sensitivity in GR_Ala610Val_ carriers, *PPARGC1A* has been shown to potently enhance transcriptional response mediated by GR (Knutti et al., 2000). We hypothesize that because GR_Ala610Val_ causes pronounced compensatory reduction in cortisol production, upregulation of *PPARGC1A* and *CEBPB* might represent a counterregulatory response, facilitating adequate glucose production under basal conditions, particularly at the nadir of the circadian rhythm when cortisol levels are lowest. Administration of low dose exogenous GC might tip the balance between available GC and response, and lead to excess production of glucose in GR_Ala610Val_ carriers. This might be further potentiated by genotype × treatment interactions. Out of the 23 DE genes involved in carbohydrate metabolism at basal condition, eight (*PFKFB3, GCK, CHGA, SLC17A3, PLA2G7, DUSP9, MFN2*, and *MMP9*) showed evidence for genotype × treatment interaction at D10. On the other hand, at higher dexamethasone doses, the genotype differences might be overridden by the vast transcriptional responses.

The most significant DE gene between Ala and Val was *PCDHB7*. *PCDHB7* belongs to a large family of protocadherins (50–60 genes in mammals), which are arranged in tandem in three closely linked gene clusters (designated alpha, beta, and gamma) (Mountoufaris et al., 2018). Besides *PCDHB7*, GR_Ala610Val_ affected, though less significantly, also expression of other members of this gene cluster including *PCDHB5, PCDHGA7, PCDHGB4*, and *PCDHGB7*. Unfortunately, in the pig the protocadherin cluster is not annotated and characterized to that extent (19 genes annotated, of which 13 were expressed in the liver) as in humans or mice, so that it is not possible to assess effect of GR_Ala610Val_ on the entire cluster. Clustered protocadherins are cell-cell adhesion proteins implicated mainly in neural circuit formation (Peek et al., 2017). While their function in neural development and related disorders, such as autism, is well studied (Mountoufaris et al., 2018), there is a lack of knowledge about their function in non-neural tissues. Outside of the nervous system clustered protocadherins might be involved in cell proliferation and cell death (El Hajj et al., 2017). Therefore, it is difficult to predict the phenotypic consequence of differential expression of *PCDHB7* and of the other clustered protocadherins in liver. Nevertheless, hepatic nervous system is involved in metabolic regulation (Jensen et al., 2013) and represents an additional potential candidate mechanism how GR_Ala610Val_ could influence liver function, also via dexamethasone action in the brain (Mizuno and Ueno, 2017). Differential expression of *FOSL2*, a marker of neuronal activation (Herdegen and Leah, 1998), might be an indication that GR_Ala610Val_ in fact altered the hepatic innervation.

Much research on clustered protocadherins was attracted by the observation that their expression is epigenetically modulated (El Hajj et al., 2017), for example by early life insults (Suderman et al., 2012). Glucocorticoids are well known for their involvement in environmental modulation of the epigenome, particularly during early life (Moisiadis and Matthews, 2014). We have shown previously that GR_Ala610Val_ downregulates HPA axis activity and glucocorticoid production in early ontogeny (Muráni et al., 2016). While epigenetic modulation of the clustered protocadherins by GR_Ala610Val_ is conceivable, our results show that their expression is not persistently altered by the mutation, but rather dynamically modulated, demonstrated by the finding that they were mainly influenced by genotype depending on dexamethasone treatment.

It should be noted that GR_Ala610Val_ is a natural variant acting in an outbred genetic background. To reduce the impact of genetic background on the analyses we used alternatively homozygous sib pairs for the experiment. Furthermore, for selected genes of interest (e.g., *PCDHB7* and *PPARGC1A*) we validated the effect of GR_Ala610Val_ in an independent cohort. Considerable linkage disequilibium typically spreads less than 20–30 kbp in pigs (Du et al., 2007). The GR locus *NR3C1* is about 130 kbp large, so that indirect responsibility of polymorphisms in linked genes for genotype differences in gene expression observed here is unlikely. This includes also potential regulatory polymorphisms of *NR3C1*, because similar to our previous studies *NR3C1* showed no significant differences between Ala and Val here.

In conclusion, in the present study, we performed the first comprehensive analysis of transcriptome responses to dexamethasone using mRNA-Seq. Our results provide novel general insights into biology and genetics of differential GC sensitivity. This knowledge may help to interpret future expression studies in farm animals, and also provide functional candidate genes to investigate, and eventually modulate, individual sensitivity to GC therapy. One important aspect addressed here was the impact of the Ala610Val substitution on GR signaling in peripheral tissues. Our results suggest adaptive changes in the regulation of glucose homeostasis in the liver, and point to *PPARGC1A* and *CEBPB* as important mediators. Moreover, our results highlight clustered protocadherins as major targets of GR_Ala610Val_, suggesting that GR hypersensitivity may influence tissue responses to GC also by changes in neuronal circuit architecture. Overall, our findings indicate that in contrast to systemic GC production, which is persistently altered, GR_Ala610Val_ modulates tissue GC responses dynamically, depending on level of circulating GC.

## Materials and Methods

### Animals, Treatment, and Sample Collection

A total of 48 purebred German Landrace piglets (each 24 alternative homozygotes AlaAla or ValVal of GR_Ala610Val_, respectively, with equal numbers of males and females per genotype) were used in the experiment. These were selected from 12 different litters (1–3 alternatively homozygous sib-pairs per litter) produced by mating heterozygous parents (4 sires and 10 dams). At the time of the experiment the piglets were 7-weeks old (weaned at 4-weeks *post natum*) and weighed on average 13.7 kg. During the experiment the piglets had free access to feed (Trede and von Pein, Itzehoe, Germany) and water. Before treatment (0 h), a blood sample (10 ml) was obtained from each animal by rapid (≤30 s) anterior *vena cava* puncture in a supine position, and collected into pre-chilled EDTA-containing tubes. Subsequently, a bolus injection of either dexamethasone (Dexatat; aniMedica, Senden, Germany) or sterile saline was administered intramuscularly into the neck muscle. Twenty piglets received saline (group C; five males and five females per each genotype), sixteen piglets were treated with 10 μg/kg dexamethasone (group D10; four males and four females per each genotype), and twelve piglets were treated with 60 μg/kg dexamethasone (group D60; three males and three females per each genotype). For an overview of sample distribution according to treatment, sex, and genotype please see Table 3. The initial 0 h blood sample collection and treatment took place at 10:00 to 12:00 AM. Three hours after the treatment (13:00 to 15:00 AM) an additional blood sample (3 h) was collected to monitor the physiological responses. After that the animals were anesthetized by an intravenous (i.v.) administration of a combination of ketamine (Ursotamin; Serum-Werk Bernburg, Bernburg, Germany) and azaperone (Stresnil; Janssen-Cilag, Neuss, Germany), and euthanized by an i.v. administration of T61 (Intervet, Unterschleißheim, Germany). Tissue samples were quickly dissected, frozen in liquid nitrogen, and stored at -80°C. The whole procedure from blood sampling to tissue preservation lasted ∼ 15 min.

**Table 3:** Summary of the study design.

	Treatment	C		D10		D60	
Genotype	Sex	Male	Female	Male	Female	Male	Female
AlaAla		5	5	4	4	3	3
ValVal		5	5	4	4	3	3

The piglets were raised, and all animal experiments were performed, at the experimental pig farm (EAS) of the Leibniz Institute for Farm Animal Biology in Dummerstorf (Germany), equipped with a surgery room where tissue collection was performed. Animal care, handling, and sample collection followed the guidelines of the German Law of Animal Protection.

### Genotyping

For genotyping of the GR_Ala610Val_ substitution (SNP *NR3C1* c.1829C > T, rs335303636) a new KASP (Kompetitive allele specific PCR) assay has been designed (LGC Genomics, Hoddesdon, United Kingdom). DNA was extracted from tissue samples using KAPA Express Extract Kit (VWR International, Darmstadt, Germany). The KASP assay was performed using KASP V4.0 2X Mastermix Low ROX (LGC Genomics) on a LightCycler 480 System (Roche, Mannheim, Germany) according to manufacturer’s instructions.

### Measurement of Metabolic Parameters

Plasma was prepared by centrifugation for 20 min at 4°C and 2000 × *g* and stored at -80°C until use. Glucose (Glu), blood urea nitrogen (BUN), and triglyceride (TG) levels were measured using a Fuji DriChem 4000i clinical chemistry analyzer (Scil, Viernheim, Germany). These three parameters were chosen to characterize dexamethasone effect on glucose, protein, and fat metabolism, respectively.

### mRNA-Seq

Total RNA was isolated from liver samples using TRI reagent (Sigma-Aldrich, Taufkirchen, Germany). DNA traces were removed by DNaseI-treatment (Baseline-Zero DNase; Biozym, Hessisch Oldendorf, Germany), and the RNA was cleaned-up using the RNA Clean&Concentrator-25 Kit (Zymo Research, Freiburg, Germany). RNA quantity was determined using a Qubit fluorometer (Thermo Fisher Scientific, Germany), and integrity was checked on an Agilent 2100 Bioanalyzer using an Agilent RNA 6000 Nano kit (Agilent Technologies, Santa Clara, CA, United States). The latter showed that all isolated RNA samples had a RIN value > 9, indicating good quality. Library preparation was performed using a TruSeq Stranded mRNA Sample Preparation kit according to the manufacture’s protocol (Illumina). The DNA libraries were quality-control assessed for fragment-size distribution using an Agilent Technologies 2100 Bioanalyzer and Agilent DNA-1000 Chip kit. DNA library concentration was quantified using a KAPA qPCR Library Quantification kit (KAPA-Biosystems). Normalized multiplexed DNA libraries of 18 pM with 0.5% spiked-in PhiX control were clonally cluster amplified on the surface of a sequencing flow-cell using the cBot system and paired-end sequenced for 2 × 101 bp using the high-output mode on a HiSeq2500 (Illumina) at the sequencing facility of the Genome Biology Institute, Leibniz Institute for Farm Animal Biology (FBN), Dummerstorf, Germany. The raw sequencing reads (fastq-formatted) were quality-checked using FastQC (version 0.11.5^1^) and were pre-processed by filtering out low quality reads with a mean Q-score < 20 and trimming adapter-like sequences using in-house scripts. This yielded on average 50.6M reads per sample. High quality paired-end reads were then aligned to the reference genome Ssrofa11.1 (Ensembl release 93) using Hisat2 version 2.1.0 (Pertea et al., 2016) resulting in 90.6% reads aligned concordantly exactly 1 time. The number of reads uniquely mapped to each gene was extracted from the HISAT2 mapping results (SAM formatted) using HTSeq version 0.8.0 (Anders et al., 2015). On average 25.96M fragments per sample were counted.

### qPCR

For quantitative real time PCR (qPCR) cDNA was synthesized using SuperScript III MMLV reverse transcriptase (Invitrogen, Karlsruhe, Germany) in a reaction containing a mixture of 500 ng random hexamers (Promega, Mannheim, Germany), 500 ng of oligo (dT)11 VN primer, and 1 μg total RNA. The qPCR reaction was performed in duplicate on a LightCycler 480 System using the LightCycler FastStart DNA Master SYBRplus Green I (Roche) kit. Specificity of the reaction was verified by melting curve analysis and agarose gel electrophoresis of amplification products. Copy numbers of target genes were calculated based on a standard curve generated by amplification of a serial dilution of a PCR fragment (10^7^-10^2^ copies) and were normalized using the expression of *RPL32, B2M*, and *TSC22D2* as reference genes. Information on primers and amplicons is summarized in Supplementary Table S8.

### Identification and Functional Annotation of Differentially Expresses Genes

Genome-wide analysis of differential gene expression was performed based on count data obtained from mRNA-Seq using the R packages edgeR (Robinson et al., 2010) and LIMMA (Liu et al., 2015; Law et al., 2016). First, weakly expressed genes were excluded from the analysis by keeping genes represented by more than 5 counts in at least 6 samples, and then scale normalization was applied using the TMM method. Supervised principal component analysis (Bair et al., 2006) was performed on normalized counts per million (cpm) using the R package dimreduce (Piironen and Vehtari, 2018). For the analysis of the effect of treatment on gene expression the linear model implemented in LIMMA included the effect of treatment (C, D10, and D60), and to adjust for individual variation also the effects of sex (male and female), and GR_Ala610Val_ genotype (AlaAla and ValVal). The effect of genotype and of the genotype × treatment interaction on gene expression was analyzed using linear contrasts comparing genotype groups within treatment, and by comparing the treatment effects between the genotype groups (each dexamethasone dose vs. control within ValVal or AlaAla), respectively. Functional annotation of differentially expressed genes was performed using Ingenuity Pathway Analysis (IPA; Ingenuity Systems, Redwood City, CA, United States). Venn diagrams were drawn using the web tool Venny 2.1^2^. Heatmap was created using the web tool ClustVIs^3^ (Metsalu and Vilo, 2015).

Weighted correlation network analysis (Langfelder and Horvath, 2008) was performed using the R package WGCNA (version 1.66). To perform co-expression analysis normalized counts per million of all differentially expressed genes due to treatment were used. To cluster/group genes within modules based on their expression profiles, the soft-thresholding power value of 4 was auto-detected using a scale free model fit threshold of 0.9. Furthermore, modules were created with a minimum module size of 50 and a minimum dissimilarity threshold between module eigengenes of 0.2. Finally, the Pearson correlation was calculated between module eigengenes and the metabolic data.

Upstream 2 kp sequences of selected sets of differentially expressed genes were retrieved from the ENSEMBL database and analyzed for over-represented regulators/binding sites using the oPOSSUM v3.0 Single Site Analysis (Kwon et al., 2012). A matched set of background sequences was generated using oPOSSUM v3.0. Motifs with *z*-score > 10 and a Fisher-score > 2.996 (*p* < 0.05) were considered significantly over-represented.

### Statistical Analysis

The effect of GR_Ala610Val_ genotype on baseline concentration of the metabolic parameters at 0h was analyzed using a linear model (GLM procedure, SAS 9.4 Software, SAS Inc., Cary, NC, United States). Besides GR_Ala610Val_ genotype (AlaAla and ValVal), the model included also sex (male, female) as a fixed effect. The effect of dexamethasone treatment on metabolic responses depending on GR_Ala610Val_ was analyzed using a linear model (GLM procedure) including the fixed effects of treatment (C, D10, and D60), sex (male and female), GR_Ala610Val_ genotype (AlaAla and ValVal), and genotype × treatment interaction, respectively. In addition, levels of the corresponding physiological parameter at 0h were included as a covariate to account for individual baseline differences. Correlation between expression data generated using mRNA-Seq (normalized CPM) and qPCR was calculated using Proc CORR (SAS 9.4). In addition, to validate treatment and genotype effects using qPCR data (log2 transformed as for mRNA-Seq data analysis) similar linear models and contrasts as described for mRNA-Seq data analysis were fitted (Mixed procedure, SAS 9.4 Software, SAS Inc., Cary, NC, United States). For the analysis of the previously published dataset the linear model included, besides genotype and sex, also sampling order as covariate.

## Ethics Statement

The Animal Care Committee of the Leibniz Institute for Farm Animal Biology and the State Mecklenburg-Western Pomerania (Landesamt für Landwirtschaft, Lebensmittelsicherheit und Fischerei) approved the experimental protocol (7221.3-1-024/16).

## Author Contributions

EM conceived the study, designed the experiments, and wrote the manuscript. EM and KW supervised and coordinated the experiments. EM and NT performed the experiments. EM, NT, and FH analyzed the data. KW and SP contributed to reagents, materials, and analysis tools. All authors critically revised and approved the final manuscript.

## Conflict of Interest Statement

The authors declare that the research was conducted in the absence of any commercial or financial relationships that could be construed as a potential conflict of interest.

## Footnotes

1 http://www.bioinformatics.babraham.ac.uk/projects/fastqc/

2 http://bioinfogp.cnb.csic.es/tools/venny

3 https://biit.cs.ut.ee/clustvis
