# Supplementary material for: Transcriptome Responses to Dexamethasone Depending on Dose and Glucocorticoid Receptor Sensitivity in the Liver
Source: Front Genet. 2019 Jun 12;10:559. doi: 10.3389/fgene.2019.00559 (PMC6582245; doi:10.3389/fgene.2019.00559)
Supplement: Supplementary file 1 [file Data_Sheet_1.pdf]

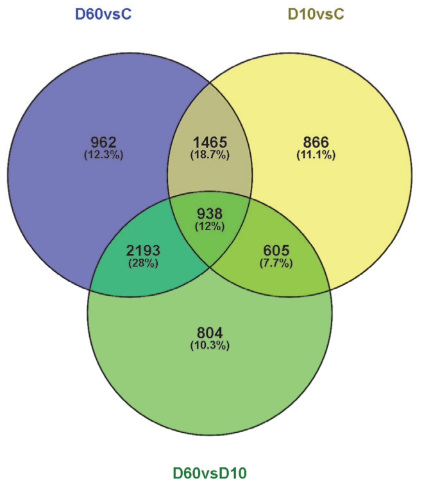

Figure S1. Venn diagram of differentially expressed genes due to treatment

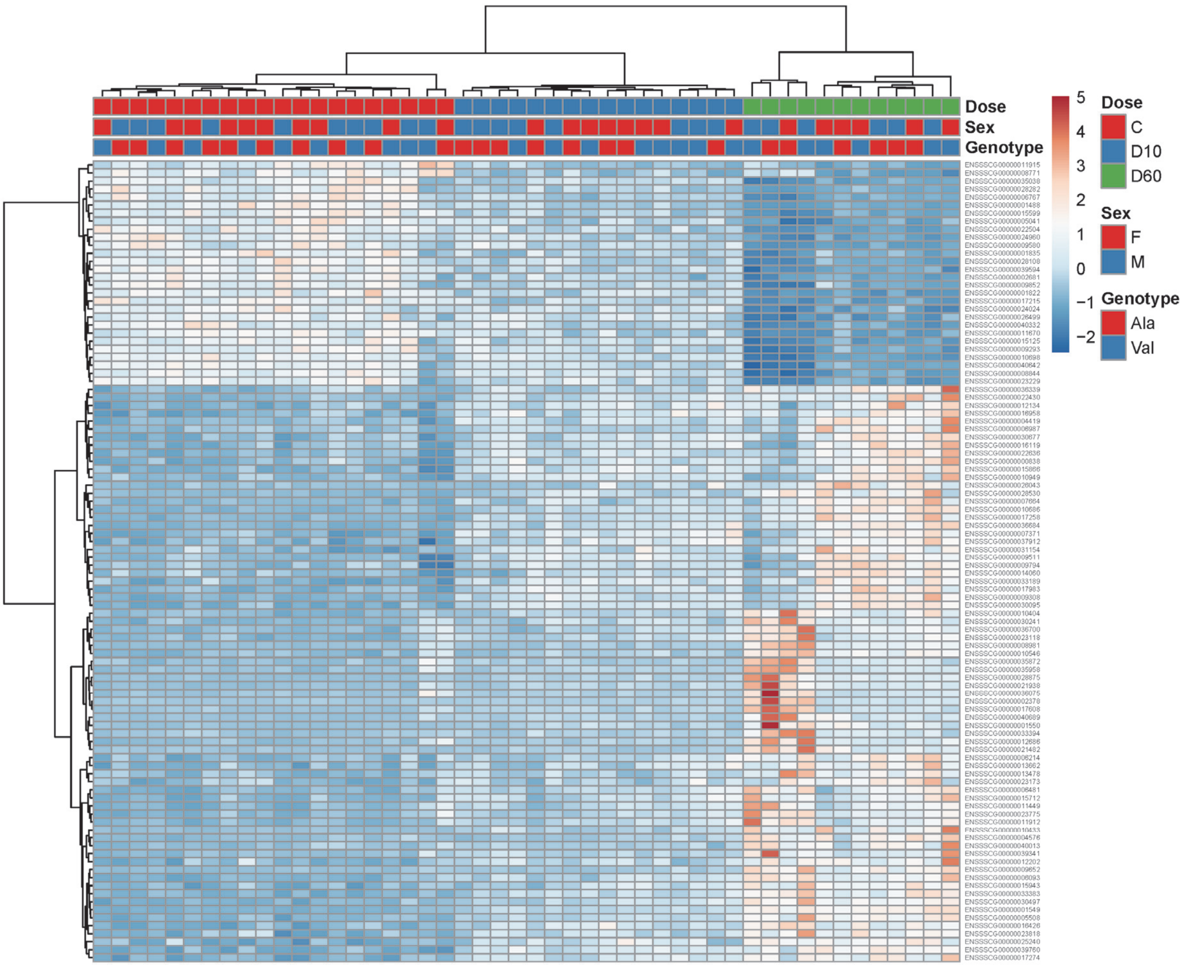

Figure S2. Clustering of top 100 genes showing dose-dependent (DD) responses

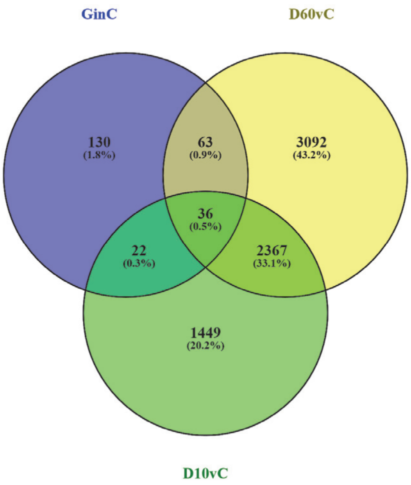

a)

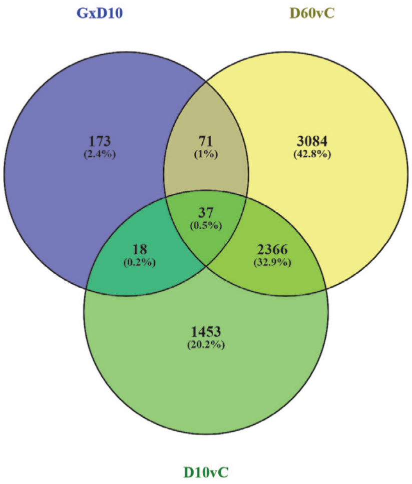

b)

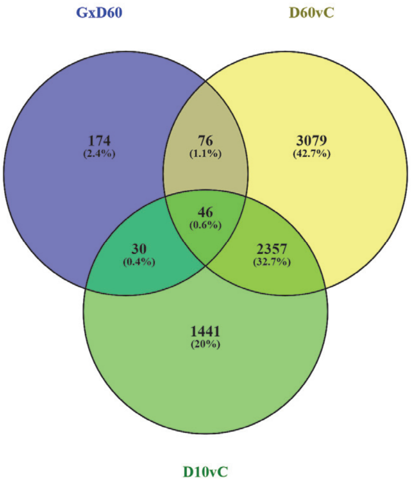

c)

Figure S3. Venn diagram comparison of genes affected by a) genotype or genotype×treatment interaction, and those responsive to dexamethasone treatment b) and c), respectively

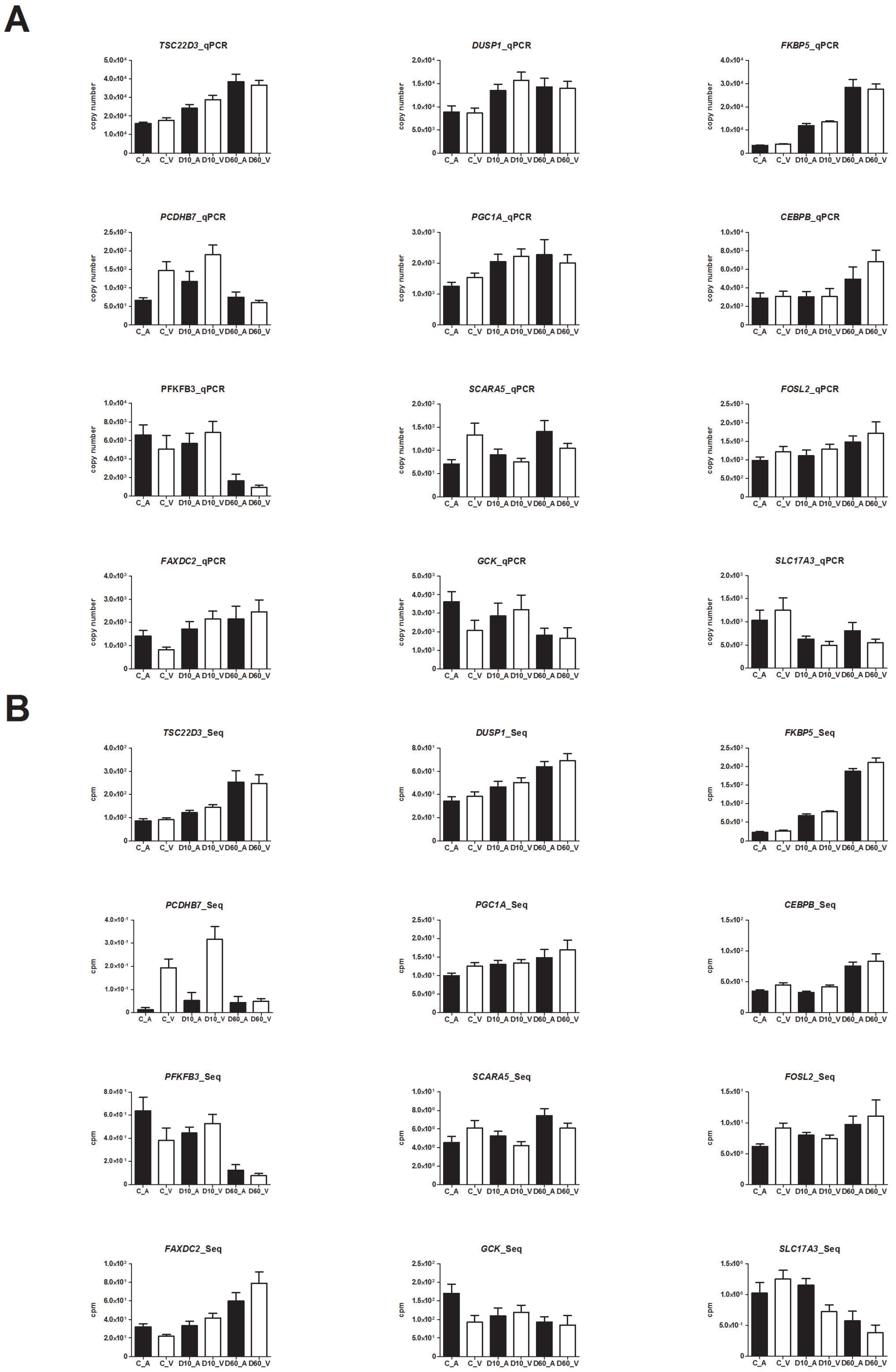

Figure S4. Expression pattern of selected genes obtained using A) qPCR and B) mRNA-Seq respectively. Black bars indicate AlaAla genotype (abbreviated A in x-axis description), white bars ValVal genotype (abbreviated V in x-axis description). C, D10, and D60 indicate saline treated group (each bar represents n=10 per genotype group within C treatment), 10 µg/kg dexamethasone treated group (each bar represents n=8 per genotype group within D10 treatment), and 60 µg/kg dexamethasone treated group (each bar represents n=6 per genotype group), respectively. Results are presented as means + standard error. CPM – counts per million.
